# A retinaculum-sparing surgical approach preserves porcine stifle joint cartilage in an experimental animal model of cartilage repair

**DOI:** 10.1186/s40634-017-0083-7

**Published:** 2017-04-17

**Authors:** Marcelo B. Bonadio, James M. Friedman, Mackenzie L. Sennett, Robert L. Mauck, George R. Dodge, Henning Madry

**Affiliations:** 1 Department of Orthopedic Surgery, University of São Paulo, São Paulo, Brazil; 2 McKay Orthopaedic Research Laboratory, University of Pennsylvania, Philadelphia, PA USA; 3 Department of Orthopaedic Surgery, Saarland University Medical Center, Homberg, Saar Germany; 4 Collaborative Research Partnership - Acute Cartilage Injury Program, AO Foundation, Davos, Switzerland

**Keywords:** Animal model, Knee, Arthrotomy, Minimally invasive, Mini-pig, Cartilage repair

## Abstract

**Background:**

This study compares a traditional parapatellar retinaculum-sacrificing arthrotomy to a retinaculum-sparing arthrotomy in a porcine stifle joint as a cartilage repair model.

**Findings:**

Surgical exposure of the femoral trochlea of ten Yucatan pigs stifle joint was performed using either a traditional medial parapatellar approach with retinaculum incision and luxation of the patella (*n* = 5) or a minimally invasive (MIS) approach which spared the patellar retinaculum (*n* = 5). Both classical and MIS approaches provided adequate access to the trochlea, enabling the creation of cartilage defects without difficulties. Four full thickness, 4 mm circular full-thickness cartilage defects were created in each trochlea. There were no intraoperative complications observed in either surgical approach. All pigs were allowed full weight-bearing and full range of motion immediately postoperatively and were euthanized between 2 and 3 weeks. The traditional approach was associated with increased cartilage wear compared to the MIS approach. Two blinded raters performed gross evaluation of the trochlea cartilage surrounding the defects according to the modified ICRS cartilage injury classification. The traditional approach cartilage received a significantly worse score than the MIS approach group from both scorers (3.2 vs 0.8, *p* = 0.01 and 2.8 vs 0, *p* = 0.005 respectively).

**Conclusion:**

The MIS approach results in less damage to the trochlear cartilage and faster return to load bearing activities. As an arthrotomy approach in the porcine model, MIS is superior to the traditional approach.

## Background

Pre-clinical animal models to assess cartilage repair are vital to advance new cartilage therapeutics to clinical trials (Reinholz et al. 2004). Large animals are considered suitable surrogate models of human disease and the porcine stifle joint is one of the preferred models for cartilage repair studies given joint mechanics, weight-bearing, and cartilage thickness are similar to those found in the human knee. The porcine stifle joint is also large enough to permit the creation of 6–8 mm diameter defects for studies either in the femoral condyle or the trochlea groove (Chiang et al. 2005; Vasara et al. 2006; Gotterbarm et al. 2008; Chu et al. 2010). The trochlea is a particularly common site for assessing the new repair techniques of focal defects. Moreover, this site is of high clinical relevance as patella-femoral defects in humans have unfavorable clinical outcomes when compared to femoral condyle defects (Kreuz et al. 2006).

In humans, exposure of the trochlear groove is most often achieved with a medial parapatellar arthrotomy. Though this necessitates the cutting of the medial retinaculum, an important stabilizer of the patella, this approach has a low complication rate in humans and is considered safe (Pongcharoen et al. 2013). However, in large animal models, it has been noted that even after a meticulous repair of the medial retinaculum, slight loosening or tightening of the retinaculum can lead to maltracking of the patella over the trochlear cartilage (Orth et al. 2013). This alteration in patella-femoral joint cartilage pressure can cause mechanical and local inflammation, and cartilage degradation, jeopardizing the interpretation of results from the cartilage repair methodology being evaluated. A recent article described a minimally invasive arthrotomy (MIS) approach in sheep that helped to prevent patellar maltracking, however, this approach has not been previously tested in a porcine model (Orth et al. 2013).

In this study, we describe outcomes following a traditional parapatellar retinaculum sacrificing arthrotomy compared to a retinaculum sparing MIS arthrotomy in a porcine model. The approach will ultimately inform future studies where focal cartilage defect repair methodology is being assessed.

## Methods

### Animals

Healthy, skeletally mature, Yucatan pigs (Sinclair Bioresources), aged between 16 and 18 months (mean body weight 68.5 kg) received water ad libitum and were fed a standard diet. All animal experiments were conducted in accordance with the national legislation on protection of animals and the National Institutes of Health (NIH) Guidelines for the Care and Use of Laboratory Animals (NIH Publication 85– 23, Rev 1985) and were approved by the University of Pennsylvania Institutional Animal Use and Care Committee.

### Surgical design

All pigs underwent monitored general anesthesia with a combination of ketamine, xylazine, and isoflourane. Surgical exposure of the femoral trochlea of the pig stifle joint was performed by the same surgeons (MB & JF) using either a traditional medial parapatellar approach with retinaculum incision and luxation of the patella (*n* = 5) or the MIS approach which spared the patellar retinaculum (*n* = 5). Four full thickness, 4 mm circular cartilage defects were created in the trochlea. All pigs were allowed full weight-bearing and full range of motion immediately postoperatively and were euthanized between 1 and 3 weeks.

Prior to surgery, pigs were placed in a supine position and the skin over the stifle joint was shaved with an electric shaver. The hind limb to be operated on was suspended by the foot. No tourniquet was applied, and limbs were aseptically prepared for surgery. The foot was covered with a sterile glove and sealed with adhesive tape, and an extremity drape was applied and fixed with adhesive tape. It is important to keep the hip and stifle joint extended during the wrapping to avoid later shifting of the drapes with mobilization of the limb during surgery.

After the pigs were euthanized, two blinded raters performed gross evaluation of the trochlea cartilage excluding the defects, according to the modified ICRS cartilage injury classification (Goebel et al. 2012). In this classification, a 0 represents intact cartilage, and a 4 represents full thickness wear.

#### Traditional surgical approach

The leg was allowed to rest without traction. A longitudinal skin incision was performed 1 cm medial to the medial border of the patella, from the level of the superior pole of the patella to the level of the tibial tuberosity. The medial skin incision was selected as this is a safer site for avoiding damage to the wound from external trauma. The prepatellar bursa was resected as needed to identify joint capsule. The joint capsule was opened with an arthrotomy from the superior pole of the patella distally to the tibial tubercle, cutting through the lateral patellar retinaculum. The lateral arthrotomy is preferred because it is easier to subluxate the patella medially in pigs. The patella was dislocated medially, exposing the trochlea. If necessary, the incision was extended into the lateral vastus to enable patellar dislocation.

Closure was performed with suturing of the medial patellar retinaculum and joint capsule with Vicryl size 0. Subcutaneous layers and skin were closed using a Vicryl size 2/0 and Biocryl 3/0, respectively.

#### MIS approach

**Fig. 1:**
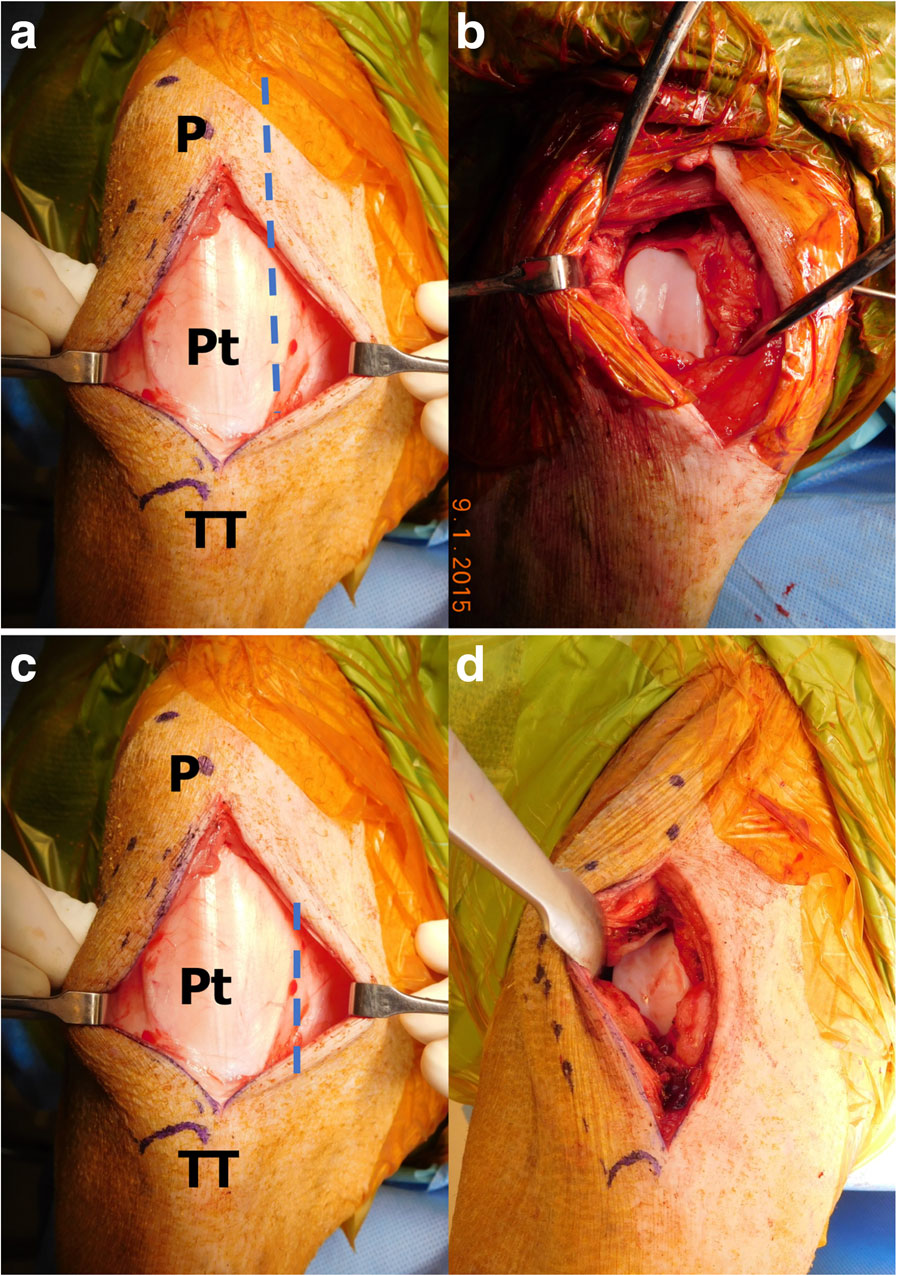
Comparison of traditional and MIS exposure of the porcine stifle joint. P represents the patella, Pt represents the patella tendon, and TT represents the tibial tubercle (**a**) Exposure of the capsule with the dotted line representing the incision of both skin and capsule needed for a traditional approach. **b** The traditional approach allows for extensive exposure of the trochlea with dislocation of the patella. **c** A much smaller incision is needed for the MIS approach. **d** The exposure of the trochlea is adequate to create cartilage defects. Note the inferior border of the patella can be seen at the superior portion of the incision and remains located

Prior to making the incision, anatomical landmarks were identified, including the tibial tubercle, patella, and patellar tendon. A straight skin incision 1 cm medial to the medial border of the patellar tendon, extending from the level of the inferior pole of the patella to the level of the tibial tubercle was made. Subcutaneous tissue was divided in line with the skin incision and the prepatellar bursa was resected as needed to identify patellar tendon edges (Fig. 1). Once adequate exposure of the patellar tendon and capsule joint was achieved the arthrotomy was made with the stifle joint in complete extension. The arthrotomy was made directly medial to the patellar tendon. It is important that the arthrotomy runs from the level of the tibial tubercle to the level of the inferior pole of the patella only in order to preserve the patellar retinaculum. Under the capsule, the fat pad was identified and incised progressively until the trochlear cartilage was visualized, taking care to not damage the cartilage. Partial resection of the fat pad facilitated the exposure of the trochlea. A small Hohmann retractor was positioned between the patellar tendon and the lateral aspect of the femoral condyle. Access to the trochlea cartilage was possible without the necessity of dislocating the patella (Fig.1). Maintaining the stifle joint in complete extension was crucial to keep the patella proximal to the trochlea, although a small degree of flexion was possible for exposure of the distal trochlea if necessary.

Closure was performed via suturing of the medial patellar retinaculum and joint capsule with Vicryl size 0. Subcutaneous layers and skin were closed using a Vicryl size 2/0 and Biocryl 3/0, respectively.

### Post-operative care

All animals were allowed full weight-bearing and full range of motion immediately postoperatively and monitored for signs of pain, distress or surgical complications on a daily basis by surgical and veterinary team. Pigs received a single dose of intravenous (IV) hydromorphone before transition to long-acting nonsteroidal anti-inflammatory drugs. Additional IV hydromorphone was delivered as needed.

### Statistical analysis

Wilcoxon rank sum test was performed to evaluate differences between scores in the two groups for each rater. The *P* value for significance was *p* < 0.05. Statistical analysis was performed using Stata 13.1 (StataCorp, College Station, Texas, USA).

## Findings

Both traditional and MIS approaches provided adequate access to the trochlea, enabling the creation of cartilage defects without difficulties. There were no intraoperative complications observed in either surgical approach. All ten pigs showed normal patellar tracking with no clinical signs of patella dislocations or wound complications during the post-operative period. The five pigs that underwent the traditional approach showed severe limping in the operative limb following the surgery for at least 72 h, and two pigs required one additional day of IV hydromorphone. Subjectively, these pigs were less interactive following surgery. Of the five pigs that underwent the MIS surgery, all five walked with minimal limp on post-operative day 1. None of the MIS surgery pigs required additional post-operative pain management and all were fully interactive by the third post-operative day.

**Fig. 2:**
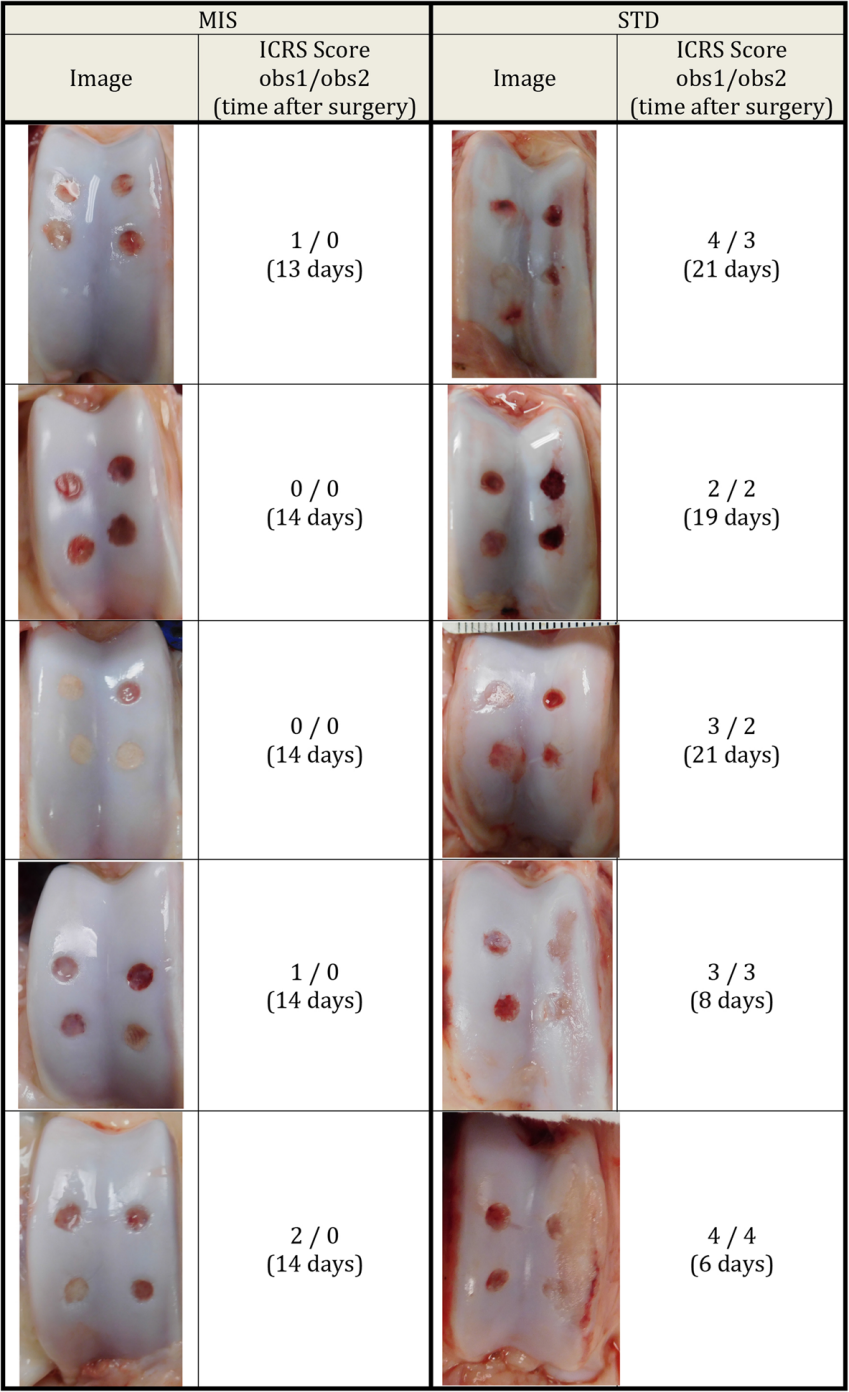
Photographs of trochlear groove and scores for Traditional vs. MIS pigs

In the post-mortem gross evaluation of the trochlear cartilage, the traditional approach group received a significantly higher score than the MIS approach group from the first scorer (3.2 vs 0.8, *p* = 0.01) and a significantly higher score from the second scorer (2.8 vs 0, *p* = 0.005) (Fig. 2). This indicated that the MIS approach groups had more intact cartilage.

## Discussion

In this study, we compared the traditional patella retinaculum sacrificing approach to a MIS retinaculum sparing approach in a porcine model. The porcine stifle joint is a common model for cartilage repair studies, reflecting many features of the human knee, including the relative sizes of articulating bones, the existence of cruciate ligaments, menisci, and a bicondylar distal femur (Reinholz et al. 2004; Gotterbarm et al. 2008; Chu et al. 2010). The trochlea in minipigs is a commonly-utilized location for articular defect models, as the larger surface area allows for multiple defects per joint. This reduces the number of animals required within a given study. Compared to other large animals such as horses, pigs have the advantage of smaller logistical requirements, are more easy handled, and are cheaper to house (Reinholz et al. 2004).

Both procedures were technically easy, safe, and yielded good exposure of the trochlea. However, the MIS approach was less invasive and decreased morbidity as compared to the traditional approach. Further benefits of the MIS approach included the preservation of important structures, including the medial patellar retinaculum, the oblique medial vastus muscle, and the patellar blood supply. The traditional approach necessitated dissection of the retinaculum, and required extensive post-operative pain management. Our finding that the traditional approach resulted in extensive postoperative morbidity is in agreement with a study by Christiansen et al., in which it was found that a traditional medial parapatellar arthrotomy in the Göttingen mini-pig resulted in greater surgical trauma and postoperative pain when compared to an alternative approach, such as the transpatellar-ligament arthrotomy (Christensen et al. 2015).

In adult pigs, the traditional approach produced some degree of gross cartilage damage in addition to the surgically-created cartilage defect. The cartilage wear appeared as a longitudinal lesion parallel to the patellar groove, suggesting that the cause of the injury was due to maltracking or patellar hyperpressure. This type of post-operative cartilage injury is not compatible with studies assessing cartilage repair methodologies such as using tissue engineered biomaterial to fill focal defects, as the entire joint environment is altered. Our observations of cartilage wear were noted when animals were euthanized within 3 weeks of surgery. It is not known if mechanical wear affected other cited studies or if mechanical damage is no longer observable at longer time points. Of note, this study only assessed adult pigs and is in contrast to our experience with juvenile pigs which do not develop additional cartilage damage following a traditional arthrotomy (Fisher et al. 2015). It is unclear what anatomic differences cause this, but the smaller weight and decreased forces on the patella may explain this different response to the same surgical approach.

There are a few potential limitations to this study. First, pigs were euthanized within 3 weeks. We therefore cannot be certain that cartilage wear does not occur at longer time points following MIS surgery. Second, there was considerable variability in the time to euthanasia within the traditional group. This could have affected the ICRS scoring results compared to the MIS group; however, we observed a similar degree of cartilage erosion at both early and late endpoints. Finally, this is a descriptive study and the sample size of 5 per group was chosen based on the overwhelming evidence to support the use of the MIS over the traditional surgical approach. Although a fully randomized and controlled study design with sample sizes determined by power analysis would be feasible, the benefits do not justify the cost, including both financial and unnecessary loss of animal life.

In adult pigs, the MIS technique preserved the native cartilage of the trochlea, resulting in a more controlled and stable joint environment that is more conducive for evaluation of cartilage repair methodology. The MIS approach may therefore be valuable in the creation of articular cartilage defects on the adult porcine femoral trochlea specific to studies of cartilage damage and regeneration.

## Conclusion

The MIS approach to the femoral trochlea it is technically easy, safe, and yields excellent exposure of the trochlea. Compared to the Traditional approach, the MIS approach results in less damage to the trochlear cartilage and faster return to load bearing activities. As an arthrotomy approach in the porcine model, MIS is superior to traditional.

## Abbreviations

IV: Intravenous
MIS: Minimally invasive surgery
